# Zika Virus Infection and Stillbirths: A Case of Hydrops Fetalis, Hydranencephaly and Fetal Demise

**DOI:** 10.1371/journal.pntd.0004517

**Published:** 2016-02-25

**Authors:** Manoel Sarno, Gielson A. Sacramento, Ricardo Khouri, Mateus S. do Rosário, Federico Costa, Gracinda Archanjo, Luciane A. Santos, Nivison Nery, Nikos Vasilakis, Albert I. Ko, Antonio R. P. de Almeida

**Affiliations:** 1 Hospital Geral Roberto Santos, Secretaria Estadual da Saúde da Bahia, Salvador, Bahia, Brazil; 2 Faculdade de Medicina da Bahia and Instituto da Saúde Coletiva, Universidade Federal da Bahia, Salvador, Bahia, Brazil; 3 Centro de Pesquisas Gonçalo Moniz, Fundação Oswaldo Cruz, Ministério da Sáude, Salvador, Bahia, Brazil; 4 Department of Epidemiology of Microbial Diseases, Yale School of Public Health, New Haven, Connecticut, United States of America; 5 Department of Pathology and Center of Biodefense and Emerging Infectious Diseases, Institute for Human Infections and Immunity, The University of Texas Medical Branch, Galveston, Texas, United States of America; Baylor College of Medicine, Texas Children's Hospital, UNITED STATES

## Abstract

**Background:**

The rapid spread of Zika virus in the Americas and current outbreak of microcephaly in Brazil has raised attention to the possible deleterious effects that the virus may have on fetuses.

**Methodology/Principal Findings:**

We report a case of a 20-year-old pregnant woman who was referred to our service after a large Zika virus outbreak in the city of Salvador, Brazil with an ultrasound examination that showed intrauterine growth retardation of the fetus at the 18^th^ gestational week. Ultrasound examinations in the 2^nd^ and 3^rd^ trimesters demonstrated severe microcephaly, hydranencephaly, intracranial calcifications and destructive lesions of posterior fossa, in addition to hydrothorax, ascites and subcutaneous edema. An induced labor was performed at the 32^nd^ gestational week due to fetal demise and delivered a female fetus. ZIKV-specific real-time polymerase chain reaction amplification products were obtained from extracts of cerebral cortex, medulla oblongata and cerebrospinal and amniotic fluid, while extracts of heart, lung, liver, vitreous body of the eye and placenta did not yield detectable products.

**Conclusions/Significance:**

This case report provides evidence that in addition to microcephaly, there may be a link between Zika virus infection and hydrops fetalis and fetal demise. Given the recent spread of the virus, systematic investigation of spontaneous abortions and stillbirths may be warranted to evaluate the risk that ZIKV infection imparts on these outcomes.

## Author Summary

The rapid spread of Zika virus in the Americas and outbreak of microcephaly in Brazil has raised attention to the possible deleterious effects that the virus may have on fetuses. We report a case of a 20-year-old pregnant woman from Salvador, Brazil whose fetus had developed hydrops fetalis, a condition where there is abnormal accumulation of fluid in the fetus, as well as severe central nervous system defects such as microcephaly and hydranencephaly. After fetal demise, ZIKV RNA was detected in central nervous system tissues and amniotic fluid. The case report provides evidence that in addition to microcephaly, there may be a link between Zika virus infection and hydrops fetalis and fetal demise. Given the recent spread of the virus, systematic investigation of spontaneous abortions and stillbirths may be warranted to evaluate the risk that ZIKV infection imparts on these outcomes.

## Introduction

The current outbreak of microcephaly has raised speculations that Zika virus (ZIKV) causes a congenital syndrome. ZIKV, a mosquito-borne flavivirus, was detected in Brazil in early 2015 [1,2] and has rapidly spread throughout the Americas [3]. A large increase in the number of newborns with microcephaly was subsequently identified in Brazil in November 2015. At present, more than 4,500 microcephaly cases have been reported [4]. ZIKV has been detected in few cases, seven in total to date, of fetuses and newborns who died shortly after birth, all of whom had ultrasound abnormalities or pathological lesions which were restricted to the central nervous system [5–7]. Herein, we report a case of a fetus that in addition to hydranencephaly, developed hydrops fetalis and fetal demise in association with congenital ZIKV infection.

## Methods

While conducting an outbreak investigation in Salvador, Brazil, we identified a patient who was referred to Hospital Geral Roberto Santos with an abnormal fetal ultrasound examination and followed during outpatient evaluations. After fetal demise and induced labor, tissues aspirates and fragments were collected by needle aspiration and thoraco-abdominal viscerotomy, respectively, since an autopsy could not be performed. RNA was extracted and tested by a ZIKV-specific reverse transcriptase-polymerase transcriptase assay (RT-PCR) [8].

## Results

We assumed the care for a 20-year-old woman (gravida 3, para 1) in the 18^th^ week of gestation whose ultrasound examination showed low fetal weight. The patient procured prenatal care during the 4^th^ gestational week in July, 2015 at which time she was found to have a negative serology for HIV, HTLV and hepatitis C viruses and positive IgG and negative IgM ELISA results for toxoplasmosis, rubella virus and cytomegalovirus. She had an uneventful course of pregnancy with a normal ultrasound evaluation at the 14^th^ gestational week. In the 18^th^ week, ultrasound examination found that the fetus had a weight three standard deviations below the mean value for gestational age.

**Fig 1:**
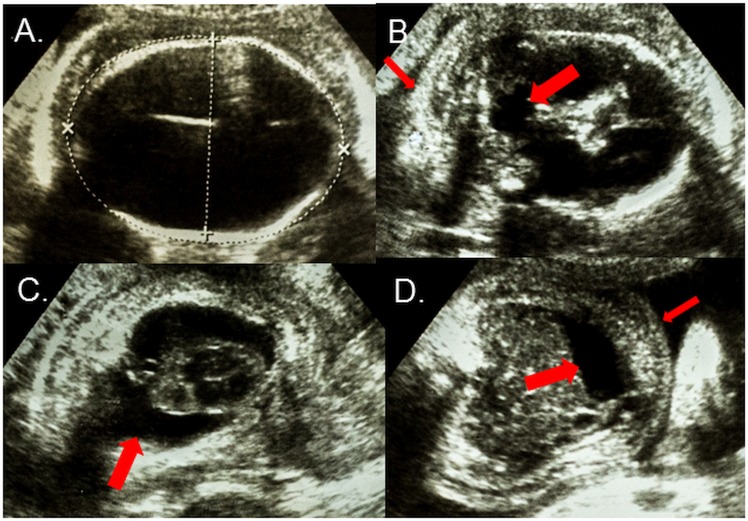
**Axial ultrasound views of the fetus at the 30^th^ gestational week showing (A) Cranium with severe microcephaly (215mm) and hydranencephaly; (B) Posterior fossa with destruction of the cerebellar vermis (wide arrow) and nuchal edema (thin arrow); (C) Thorax with bilateral pleural effusions (arrow); and (D) Abdomen with ascites (wide arrow) and subcutaneous edema (thin arrow).**

On referral, the patient did not report an episode of rash, fever, or body pain or receiving a diagnosis for zika, chikungunya or dengue virus infection during the pregnancy. She denied a family history of an illness suggestive of a Zika virus infection or congenital disorders. Her clinical evaluation was unremarkable. Ultrasound examinations performed at the 26^th^ and 30^th^ gestational weeks showed microcephaly, hydranencephaly with minimal residual cortical parenchyma (Fig 1, Panel A), intracranial calcifications and destructive lesions of posterior fossa (Fig 1, Panel B). The examinations were also significant for the findings of hydrothorax, ascites and subcutaneous edema (Fig 1, Panels C and D).

An induced labor was performed when ultrasound examination in the 32^nd^ gestational week showed fetal demise and delivered a female fetus with a weight of 930g and signs of microcephaly and arthrogryposis. We obtained ZIKV-specific RT-PCR amplification products from extracts of cerebral cortex, medulla oblongata and cerebrospinal and amniotic fluid. Analysis of extracts of heart, lung, liver, vitreous body of the eye and placenta did not yield detectable products. Amplification products mapped within the NS5 gene of ZIKV strains belonging to the Asian lineage, with closest relationship to sequences from French Polynesian and Surinamese strains. The patient gave consent to have her case details published.

## Discussion

Attention has focused on the deleterious effects that the ZIKV may have on fetuses due to the rapid global spread of virus and the current outbreak of microcephaly in Brazil. ZIKV has been detected in a small number of cases of fetuses and newborns with microcephaly who have been identified during the outbreak [5–7]. This case report of a fetus provides additional evidence for the link between ZIKV infection and microcephaly. Furthermore, it serves as an alert to clinicians that in addition to central nervous system and ophthalmological manifestations [6,7,9], congenital ZIKV infection may cause hydrops fetalis and fetal demise.

Since the majority (73%) of ZIKV infections are asymptomatic [10], it is likely that exposures in pregnant women, such as in the case of our patient, often go unnoticed. We could not document acute infection in the mother and discard the possibility, albeit unlikely, that the severe manifestations, observed in this case, was caused by another process and intrauterine ZIKV infection occurred afterwards. The first indication of an abnormal pregnancy was the ultrasound finding of intrauterine growth retardation in the 18^th^ gestational week. The more plausible explanation is that asymptomatic exposure of the mother, prior to this date and likely in the 1^st^ trimester, caused an intrauterine infection which in turn, resulted in hydranencephaly and hydrops fetalis in the fetus.

The finding of an association between ZIKV infection and hydrops fetalis suggests that the virus may cause damage to tissues in addition to the fetal central nervous system. Recent autopsy studies found that histopathologic findings and detection of ZIKV in newborns and fetuses with microcephaly were limited to the brain and in some cases, placenta [6,7], indicating that the virus, unlike common congenital viral infections, exhibits tropism to a limited range of tissues. We detected ZIKV RNA in the central nervous system and amniotic fluid and not in heart, lung, liver or placenta, yet our findings were limited by the sampling procedure and lack of histopathological analysis of tissues. The mechanism by which ZIKV may cause hydrops fetalis therefore remains speculative.

We cannot extrapolate from this single case the overall risk for developing hydrops fetalis and fetal demise among pregnant women exposed to the virus. The strain detected in this case of fetal demise appears to be the same as the epidemic strain that has spread across the Americas and Caribbean [6,11]. Given that large numbers of pregnant women in the region have been or will be exposed to this strain, systematic investigation of spontaneous abortions and stillbirths may be warranted to evaluate the risk that ZIKV infection imparts on these outcomes.
